# Radial Artery Pseudoaneurysm From a Squirrel Bite

**DOI:** 10.7759/cureus.46080

**Published:** 2023-09-27

**Authors:** Lucas Winter, Tahla Chaudhry, James L Wilson, Joshua Walker, Derrick Huang

**Affiliations:** 1 Emergency Medicine, University of Central Florida, Ocala, USA; 2 Emergency Medicine, HCA Florida Ocala Hospital, Ocala, USA; 3 Emergency Medicine, University of Central Florida, Orlando, USA

**Keywords:** pseudoaneurysm, forearm radial artery, emergency medicine, animal bite, squirrel

## Abstract

Radial artery pseudoaneurysm is a rarelimb-threatening complication that occurs from vascular procedures and direct trauma. We present a rare case of a 74-year-old female who presented to the emergency department with a squirrel bite to her right wrist. Although initially benign-appearing, computed tomography angiography of the right upper extremity showed a pseudoaneurysm at the distal radial artery. The patient was successfully treated with careful compression and rapid resolution was confirmed with an arterial right upper extremity ultrasound that visualized a formed thrombus. Emergency providers should have a high index of suspicion for radial artery pseudoaneurysms in the setting of animal bites to the wrist.

## Introduction

Radial artery pseudoaneurysm is a rare limb-threatening complication typically associated with vascular procedures and direct trauma [1]. This pathology may be a rare complication of hand and wrist injuries, which encompass over 12% of all traumatic injuries in the United States and may lead to significant disability [2]. Pseudoaneurysms involve extravasation of arterial blood into surrounding tissue with a communication between the affected artery and tissue cavity that results in a false aneurysm without involvement of the three layers of the arterial wall [1]. The femoral and radial arteries are the most common sites of pseudoaneurysms [1]. The development of pseudoaneurysms is most typically associated with procedural complications, such as coronary artery catheterization, arterial puncture for blood gas analysis and hemodynamic monitoring, and direct trauma. This pathology presents as a pulsatile mass that may also have a bruit, localized skin tenderness, and discoloration [1,3-5]. Misdiagnosis of this injury can result in delayed bleeding and complications such as eventual distal embolism with resultant ischemia, carpal tunnel syndrome, rupture, bone erosion, and peripheral neuropathy [5-7]. Although extremity injuries involving animal bites are common, pseudoaneurysm formation is rare and has been associated with snake and cat bites in other case reports [1,8]. Here we present a unique case of a radial artery pseudoaneurysm in an elderly woman after a squirrel bite to her wrist.

## Case presentation

A 74-year-old female with a past medical history of hypertension and hypothyroidism presented to the emergency department (ED) with an animal bite occurring just prior to arrival. The patient reported her pet squirrel bit her in the right forearm near the wrist as she was trying to take tissues away from the pet. She struck the squirrel, provoking the attack. Emergency medical technicians (EMT) wrapped the patient's wrist in gauze to tamponade the pulsatile bleeding identified on scene. The patient denied right upper extremity numbness, tingling, and weakness. The patient took a daily aspirin and denied use of anticoagulation. The squirrel did not have a vaccination history and had been peaceful in the past. The patient did not report a past history of surgeries requiring endovascular intervention.

On exam, the patient had a pulse of 73 beats per minute and a blood pressure of 197/81 mmHg that improved to 137/81 mmHg after intravenous morphine for pain control. On extremity exam, there was a 1 cm curvilinear laceration at the right radial aspect of the distal lateral volar forearm with blood oozing out of the laceration site with surrounding ecchymosis. No pulsatile bleeding was noted on arrival after gauze was placed to tamponade the injury by EMTs. There was a strong radial pulse with a capillary refill of less than 2 seconds. There was a full range of motion of all fingers. There was a strong triphasic Doppler signal present in the right ulnar, palmar arch, and radial artery distal to the injury. A pressure dressing was placed after irrigation with normal saline.

**Figure 1:**
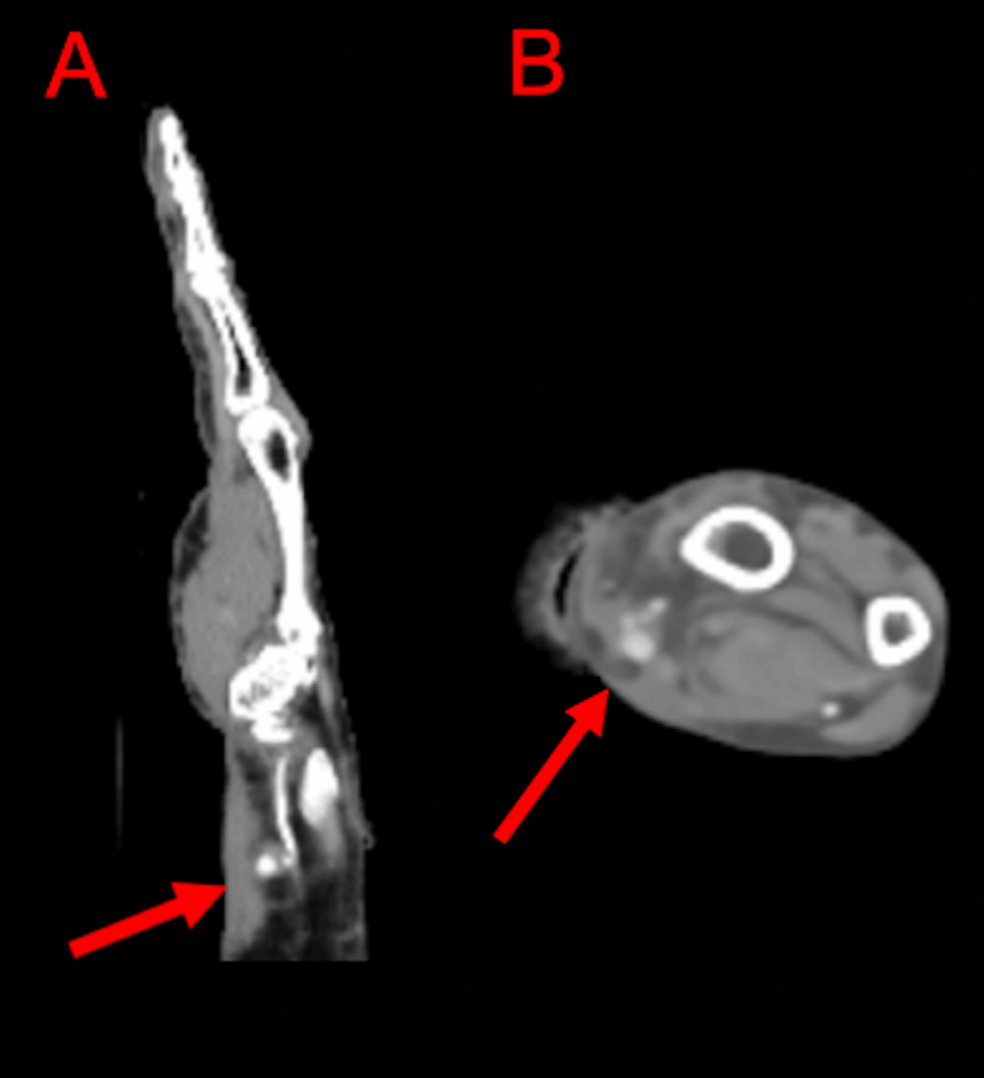
Computed tomography with angiography of the right upper extremity Sagittal (A) and transverse (B) views showing a 5 mm pseudoaneurysm adjacent to the distal radial artery with a linear tract of contrast extending back to radial artery about 3.1 cm proximal to the distal margin of the radial styloid with normal distal opacification.

**Figure 2:**
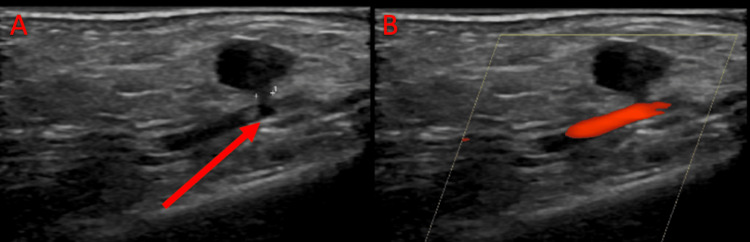
Bedside ultrasound of the right upper extremity Longitudinal view showing a (A) thrombosed pseudoaneurysm at the radial artery measuring 1.0 x 0.8 x 0.6 cm with a (B) 2 mm neck without flow on Doppler imaging.

Tetanus vaccine booster was updated and doxycycline was administered in the ED. The patient refused rabies vaccination or immunoglobulin treatment. Her lab work, including coagulation profile, was unremarkable. Due to suspicion of a significant arterial injury on history and disproportionate tenderness on clinical examination, computed tomography angiography (CTA) of the right upper extremity was performed and showed a 5 mm pseudoaneurysm adjacent to the distal radial artery with normal distal opacification of the right radial artery (Figure 1). Vascular surgery was consulted and the patient was admitted overnight. She had an arterial right upper extremity ultrasound the next day, which showed a pseudoaneurysm in the radial artery without flow on Doppler imaging along with a visualized thrombus (Figure 2). Given stability in size and completed thrombosis of her pseudoaneurysm in addition to clinical improvement in pain and lack of neurological signs and symptoms, the patient was discharged with antibiotic prophylaxis. The patient did not require outpatient vascular follow-up.

## Discussion

Radial artery pseudoaneurysm is rarely caused by an animal bite and consequences of a missed diagnosis can involve significant complications such as delayed bleeding and eventual distal embolism, limb ischemia, spontaneous rupture, and peripheral nerve dysfunction [5,6]. Risk factors for the development of pseudoaneurysms include advanced age, female gender, obesity, collagen tissue disorders, smoking, low platelet levels, use of antiplatelet or anticoagulant agents, traumatic injury, and arterial procedures [5]. Although CTA is the gold standard for diagnosis when arterial injury is suspected in the setting of pulsatile arterial bleeding, expanding hematoma, loss of pulse, and neurological signs and symptoms, ultrasound can both be used to screen for the pathology and, as in our case, assess for resolution or spontaneous thrombosis, which can facilitate appropriate disposition [5]. In obese patients, ultrasound may not be as beneficial [5]. As in our case, a clinical assessment involving both history of pulsatile bleeding and disproportionate tenderness on exam is essential, especially for smaller pseudoaneurysms caused by squirrels that can be easily missed with potential complications such as re-bleeding and eventual limb ischemia. This is especially important if compression was initiated on-scene, which can potentially hide pulsatile bleeding on exam in the ED when the patient arrives.

Treatment of pseudoaneurysms range from thrombin injection and vascular repair to ultrasound-guided or simple compression with observation [1,5,7,9,10]. Indications of vascular repair include an enlarging hematoma resulting in claudication, neuropathy, limb ischemia, uncontrolled active bleeding, and skin necrosis [9,10]. Smaller pseudoaneurysms with a size of 3 cm or less are more likely to spontaneously thrombose whereas larger pseudoaneurysms or smaller pseudoaneurysms that continue to enlarge may require more invasive treatment [5]. In our case, simple compression with careful avoidance of iatrogenic ischemia from overcompression was effective and resulted in rapid thrombosis confirmed on ultrasound (Figure 2). In many other cases, conservative management may take several weeks [9,10]. However, this approach avoids the complications of surgical repair, such as infection, wound dehiscence, rebleeding, and venous thrombosis [5].

## Conclusions

Radial artery pseudoaneurysms are a rare complication of iatrogenic trauma from arterial access. Even more rare are pseudoaneurysms occurring secondary to animal bites. Given that these injuries can be limb-threatening, providers should have a high index of suspicion for radial artery pseudoaneurysms in the setting of animal bites and similar injuries, as compression initiated on-scene for smaller bite injuries may temporarily hide pulsatile bleeding on exam in the ED. Furthermore, small, uncomplicated radial artery pseudoaneurysms secondary to an animal bite can be effectively managed with simple compression and oral antibiotics with outpatient follow-up.
